# Practical Person-Fit Assessment with the Linear FA Model: New Developments and a Comparative Study

**DOI:** 10.3389/fpsyg.2016.01973

**Published:** 2016-12-27

**Authors:** Pere J. Ferrando, Andreu Vigil-Colet, Urbano Lorenzo-Seva

**Affiliations:** Research Center for Behavior Assessment, Department of Psychology, Universitat Rovira I VirgiliTarragona, Spain

**Keywords:** person-fit statistics, linear factor analysis, mean-squared person-fit indices, personal correlation, outliers detection

## Abstract

Linear factor analysis (FA) is, possibly, the most widely used model in psychometric applications based on graded-response or more continuous items. However, in these applications consistency at the individual level (person fit) is virtually never assessed. The aim of the present study is to propose a simple and workable approach to routinely assess person fit in FA-based studies. To do so, we first consider five potentially appropriate indices, of which one is a new proposal and the other is a modification of an existing index. Next, the effectiveness of these indices is assessed by using (a) a thorough simulation study that attempts to mimic realistic conditions, and (b) an illustrative example based on real data. Results suggest that the mean-squared *lico* index and the personal correlation work well in conjunction and can function effectively for detecting different types of inconsistency. Finally future directions and lines of research are discussed.

## Introduction

When used for item analysis and individual scoring purposes, the standard factor-analysis (FA) model can be viewed as a linear item response theory (IRT) model intended for continuous scores (e.g., Ferrando, 2009). In practice it is generally used with discrete item scores and in these cases it can be only approximately correct. However, for graded-response or more continuous item formats, the linear FA approximation has proved to be reasonably good in many conditions that can be found in practice (Hofstee et al., 1998; Ferrando, 2009; Rhemtulla et al., 2012; Culpepper, 2013; Ferrando and Lorenzo-Seva, 2013). Furthermore, in comparison to the theoretically more appropriate nonlinear models, linear FA has the non-negligible advantages of simplicity, and robustness (e.g., Briggs and MacCallum, 2003; Ferrando and Lorenzo-Seva, 2013).

The appropriateness of the FA model is usually assessed by conducting an overall goodness-of-fit investigation based on the entire dataset (e.g., Reise and Widaman, 1999). Model-data fit, however, can also be assessed at the individual-level, by considering the responses of each individual across the set of test items. This level of assessment, which is usually known as “person fit,” is almost always neglected in psychometric FA applications, and is the topic of the present article.

Person-fit analysis refers to a variety of indices and procedures aimed at assessing the fit of each individual score pattern to the psychometric model fitted to the data (see e.g., Meijer et al., 2015). This type of assessment is generally sequential (e.g., Rupp, 2013; Conijn et al., 2015; Ferrando, 2015; Meijer et al., 2015), and the simplest schema is two-stage. In the first stage, a global or practical index is used to flag potentially inconsistent respondents without specifying the kind of inconsistency. In the second stage, a more specific analysis is carried out in order to ascertain the sources and effects of misfit in those patterns that are flagged as potentially inconsistent. Here we shall only consider practical indices to be used in the first stage.

Person-fit assessment is important for various reasons (see e.g., Reise and Widaman, 1999; Meijer et al., 2015) but mainly for a practical validity reason: if a response pattern is not well explained by the model, there is no guarantee that the score assigned to this pattern will adequately reflect the “true” trait level of the individual. So, this score cannot be validly interpreted. This compelling reason requires individual response patterns to be routinely checked so that invalid test scores can be detected (e.g., International Test Commission, 2014; Tendeiro and Meijer, 2014). In IRT applications, however, this recommendation is far from common practice (Meijer et al., 2015), and practical person-fit indices appear to be used routinely only in Rasch-based applications, possibly because they have been implemented and provided as standard output in these computer programs ever since they have been available (Wright et al., 1979; Smith, 1986).

The main contention of this article is that routine FA-based person fit assessment will only become (hopefully) common practice if (a) a clear proposal based on simple, effective and easily interpretable practical indices is made, and (b) this proposal is implemented in a free, user-friendly program that is easily available.

In principle, the procedures considered here could be (a) applied to both unidimensional and multidimensional solutions, and (b) used in both typical-response (personality and attitude) and ability measurement (e.g., Clark, 2010). For the moment, however, we shall focus only on unidimensional solutions intended for typical-response items. As for the first restriction, the unidimensional model is the simplest and the most univocally interpretable, and, therefore, is expected to lead to clearer results regarding person-fit assessments (e.g., Conijn et al., 2014). As for the second, most of the existing measures based on graded or more continuous items are typical-response (e.g., Ferrando, 2009).

## Review of basic FA results

Consider a questionnaire made up of *n* items with (approximately) continuous responses that intends to measure a single trait or common factor θ. For a person *i* who responds to an item *j*, the linear FA model is:

(1) $$\begin{array}{l} X_{ij}=\mu_{j}+\lambda_{j}\theta_{i}+\varepsilon_{ij} \end{array}$$

where: *X*_*ij*_ is the observed item score, μ_*j*_ is the item intercept, λ_*j*_ the item loading, ϵ_*ij*_ the measurement error, and θ is scaled in a *z*-score metric (mean 0 and variance 1). For fixed θ, the item scores are distributed independently (local independence), and the conditional distribution is assumed to be normal, with mean and variance given by

(2) $$\begin{array}{l} {\hat{X}}_{ij}=E\left(X_{j}\;|\;\theta_{i}\right)=\mu_{j}+\lambda_{j}\theta_{i}\;;\;Var\left(X_{j}|\theta \right)=\sigma_{\varepsilon j}^{2} \end{array}$$

If the item and person parameters in Equations (1) and (2) are known, it then follows that the standardized residual:

(3) $$\begin{array}{l} z_{ij}=\left(\frac{X_{ij}-{\hat{X}}_{ij}}{\sigma_{\varepsilon j}}\right) \end{array}$$

is a value drawn at random from the standard normal distribution. By the local independence principle, it then follows that the sum:

(4) $$\begin{array}{l} S_{i}=\overset{n}{\underset{j}{\sum }}{z^{2}}_{ij} \end{array}$$

is distributed as χ^2^ with *n* degrees of freedom. So, *E*(*S*_*i*_) = *n*, and Var(*S*_*i*_) = 2*n*.

In most practical applications, neither the structural parameters (μ_*j*_,λ_*j*_, and $\sigma_{\epsilon j}^{2}$) nor the “true” trait levels θ_*i*_ are known, and they have to be estimated. We shall assume here that model (1) is fitted using a standard two-stage procedure (McDonald, 1982). In the first stage (item calibration), the structural (item) parameters are estimated. In the second stage (scoring), the item estimates are taken as fixed and known, and used to obtain trait estimates or factor scores for each individual. We shall further assume that the individual trait estimates are maximum likelihood (ML) estimates, given by

(5) $$\begin{array}{l} {\hat{\theta }}_{i}\left(ML\right)=\frac{\overset{n}{\underset{j}{\sum }}\frac{\lambda_{j}\left(X_{ij}\;-\;\mu_{j}\right)}{{\sigma_{\varepsilon j}}^{2}}}{\overset{n}{\underset{j}{\sum }}\frac{{\lambda_{j}}^{2}}{{\sigma_{\varepsilon j}}^{2}}} \end{array}$$

In FA terminology, the estimates in Equation (5) are known as Bartlett's weighted least squares factor scores (e.g., McDonald, 1982).

## Overview of the selected indices and rationale

**Figure 1:**
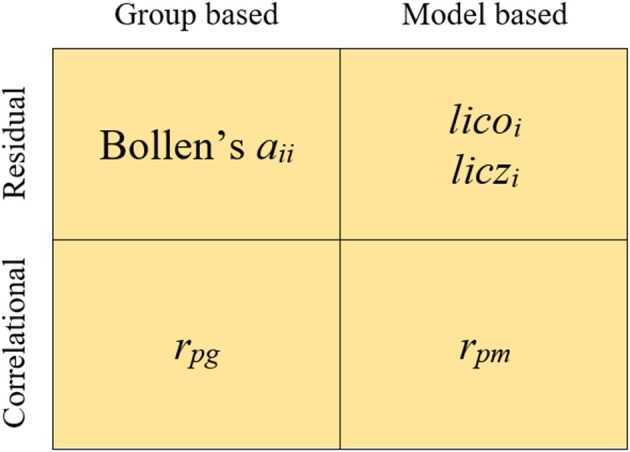
**Indices used in the study**.

The indices we shall consider in the study fall into four different categories which arise when two different criteria are combined. The resulting categories and indices are summarized in Figure 1.

The first criterion distinguishes between model-based (MB) or parametric vs. model-free or group-based (GB) indices. In MB indices, the information provided by the parameter estimates of the model is used to assess person fit. In the case of FA this information refers to (a) the item parameter estimates and (b) the individual trait level estimate or factor score. In contrast, the GB indices use only the information provided by the responses of the group of individuals which is assessed. So, the fit of the response pattern is assessed with respect to the majority of response patterns in the group (e.g., Tendeiro and Meijer, 2014).

Because MB indices use more information than GB indices they should be more powerful. In simulation studies, however, it is not unusual for GB indices to outperform their theoretically superior counterparts (Karabatsos, 2003; Tendeiro and Meijer, 2014; Meijer et al., 2015). This result does have some plausible explanations. First, the presence of some inconsistent respondents might distort the structural (i.e., item) estimates (Nering, 1997). Second, the same response vector that is used to obtain the trait estimate is then used to assess person misfit. So, if the response vector does not fit, the inconsistency is likely to bias the trait estimate and this bias, in turn, will distort the MB person-fit value in the direction of making the response vector appear less inconsistent than it really is (Karabatsos, 2003; Armstrong et al., 2007). The source of this second problem is, indeed, that the true trait levels are unknown, so estimates (ML in our case) are used in their place. In general, the closer the estimates are to the true values, the more effective the MB indices will be at detecting inconsistencies (Reise, 1995). However, to one extent or another, trait estimates are unreliable and indeterminate (i.e., the problem of factor indeterminacy, see Guttman, 1955), and the more unreliable and indeterminate they are, the less effective the MB indices based on them are expected to be.

The second criterion in Figure 1 distinguishes between residual vs. correlational indices. Residual indices are generally mean-squared measures that assess the discrepancies between the observed and the expected (from the model estimates or from the group responses) response vectors. Correlational indices are based on the product-moment correlation between the observed-expected vectors.

The relations between residual and correlational indices can be discussed by using some basic concepts from profile analysis. The residual indices that we shall consider here are *D*^2^–type indices (Cronbach and Gleser, 1953), based on the squared distance between the observed and the expected vectors. So, they simultaneously consider differences in elevation (score means), scatter or dispersion (score standard deviations) and shape (mainly rank ordering agreement between observed and expected scores). In contrast, correlational indices are only affected by differences in shape. So, in principle residual indices should be more powerful than correlational indices because they use more information from the data. Again, however, the simpler correlational indices have performed surprisingly well in some simulation studies (Rudner, 1983).

## Residual-based indices

### GB indices

In the more general field of outlier detection, Bollen (1987) proposed a model-free residual statistic which is, essentially, a scaled Mahalanobis distance based on an unstructured covariance matrix (e.g., Yuan et al., 2004). Denote by **Z**, of dimension *N* × *n*, the matrix containing all the person × item scores written as deviations from the variable means. Next, define the *N* × *N* **A** matrix as

(6) $$\begin{array}{l} A=Z{\left(Z\prime Z\right)}^{-1}Z\prime \end{array}$$

The elements a_ii_ in the main diagonal of **A** are Bollen's person-fit indices for the *i* individual. These elements measure the distance of the response vector of individual *i* from the means for all of the items. They tend to flag as potentially inconsistent those cases that sit far away from the center of the data. In terms of interpretation they have two interesting properties: first, they are scaled to provide values in the range 0–1. Second, their average value is *n/N* and this is a reference for judging the magnitude of a_ii_. The main shortcoming is that an individual with an extreme trait level that responds consistently with the FA model may be flagged as potentially inconsistent with this index.

### MB indices

Several indices have been proposed in this category (Bollen and Arminger, 1991; Yuan et al., 2004). Here, we shall consider an index proposed by Ferrando (2007) denoted here as *lco*. It is the sum of the squared residuals in Equation (4) evaluated by using the ML trait estimate in Equation (5) instead of the unknown “true” trait level.

(7) $$\begin{array}{l} lco_{i}={\overset{n}{\underset{j}{\sum }}\frac{\left(X_{ij}-\mu_{j}-\lambda_{j}{\hat{\theta }}_{i}\left(ML\right)\right)}{{\sigma^{2}}_{\varepsilon j}}}^{2}. \end{array}$$

Because a minimum-chi-square trait estimate is used as a substitute for θ, it follows that, if the model is correct and under the null hypothesis that all the respondents are consistent, the distribution of *lco* is expected to be χ^2^ with *n-1* degrees of freedom. So, the expected value of *lco* is *n-1* and its variance is *2(n-1)*. Conceptually *lco* measures discrepancies between an individual pattern of observed scores and the pattern which would be expected from the FA model given the trait estimate for this individual. So, large *lco* values indicate non-fitting response patterns.

Our real-data applications based on *lco* suggest that the index is of practical interest, but they have also revealed a problem of over-sensitivity to unexpected responses in items of good quality (i.e., with a small residual variance). This result can be anticipated by inspecting Equation (7) and is well documented in Rasch analysis, in which discrepancy indices of the form Equation (7) are labeled as “outfit” statistics [meaning outlier-sensitive fit (e.g., Wright and Masters, 1982; Smith et al., 1998)].

In Rasch-based measurement, weighted discrepancy indices labeled as “infit” statistics, have been proposed to counteract the over-sensitivity problem discussed above (Wright and Masters, 1982; Smith et al., 1998). In the same spirit, we propose here a new FA-based weighted statistic which is defined as

(8) $$\begin{array}{l} lico_{i}=\left(\frac{n}{n-1}\right)\frac{{\overset{n}{\underset{j}{\sum }}\left(X_{ij}-\mu_{j}-\lambda_{j}{\hat{\theta }}_{i}\left(ML\right)\right)}^{2}}{\overset{n}{\underset{j}{\sum }}{\sigma^{2}}_{\varepsilon j}}. \end{array}$$

To derive the mean and variance of *lico*, consider first the simple case in which the trait levels are known. In this case, Equation (8) could be written as (see Equations 2, 3):

(9) $$\begin{array}{l} lico_{i}=\left(\frac{n}{n-1}\right)\frac{\sum_{j}^{n}{\sigma^{2}}_{\varepsilon j}{z^{2}}_{ij}}{\sum_{j}^{n}{\sigma^{2}}_{\varepsilon j}}=\sum_{j}^{n}w_{j}{z^{2}}_{ij}, \end{array}$$

i.e., a linear combination of independent χ^2^ variables (${\text{z}}_{j}^{2}$) each of which has one degree of freedom. So, *E*(${\text{z}}_{j}^{2}$) = 1 and *Var*(${\text{z}}_{j}^{2}$) = 2. By considering next the loss of one degree of freedom when ${\hat{\theta }}_{i}\left(ML\right)$ is used instead of the unknown θ_*i*_, the mean and variance of *lico* are found to be

(10) $$\begin{array}{l} E\left(lico_{i}\right)=1\\ Var\left(lico_{i}\right)=\left(\frac{n}{n-1}\right)\frac{2\overset{n}{\underset{j}{\sum }}{\sigma^{4}}_{\varepsilon j}}{{\left(\overset{n}{\underset{j}{\sum }}{\sigma^{2}}_{\varepsilon j}\right)}^{2}}. \end{array}$$

Overall, *lico* is a weighted mean-squared statistic which has unit expectation under the null hypothesis of consistency. As in the case of *lco*, large values (in this case larger than the unit reference value) suggest inconsistency. As for cutoff values, in Rasch measurement conventional values of about 1.3–1.5 are generally used for judging potential inconsistency based on this type of statistics (e.g., Wright and Linacre, 1994). However, Equation (9) shows that the expected variance of *lico* (and, therefore, its expected range of values) mainly depends on test length. To see this point more clearly, consider that in the case of parallel items, with equal residual variances, the variance term in (9) reduces to *2/(n-1)*, which is indeed the variance of *lco/(n-1)*. For weighted discrepancy indices based on the Rasch model, Smith et al. (1998) suggested more refined cutoff values that take into account this dependence. They are given by:

(11) $$\begin{array}{l} critical\;value=1+\frac{2}{\sqrt{n}}. \end{array}$$

The appropriateness of this cutoff for the present proposal will be assessed in both the simulation study and the illustrative example.

An alternative possibility in terms of interpretation and cutoff values is to obtain a standardized version of *lico* that can be interpreted as a normal deviate. To do so, we shall consider again the simple case in which the trait levels are known and use the linear-composite expression (9). Jensen and Solomon (1972) found that combinations of this type can be closely approximated to the standard normal by using a Wilson-Hilferty cube-root transformation (Wilson and Hilferty, 1931). Our contention is that this approximation will also be close enough to the normal when ML trait estimates are used instead of unknown true levels. If it is, the new standardized person-fit statistic we propose could be computed as

(12) $$\begin{array}{l} licz_{i}=\left(lic{o_{i}}^{1/3}-1\right)\left(\frac{3}{\sqrt{Var}\left(lico_{i}\right)}\right)+\left(\frac{\sqrt{Var}\left(lico_{i}\right)}{3}\right). \end{array}$$

In principle, the theoretically-derived *Var(lico*_*i*_*)* is given in Equation (10). However, our preliminary simulation studies suggest that, while the empirical mean value of *lico* is usually quite close to the expected unit value, the empirical variance may be different from the theoretical variance in Equation (10). If it is, the use of the latter is expected to lead to differences between Equation (12) and the reference simulation. To address this problem, we propose to empirically estimate the variance of *lico* by using simulation procedures, and then use this empirical estimate in Equation (12). If it works properly, this combined theoretical-empirical procedure has the advantage that the index can still be interpreted as a normal deviate, with its familiar associated cutoff values that do not depend on test length.

### Correlation-based indices

#### Group-based indices

Fowler (1954) and Donlon and Fischer (1968) proposed using the correlation between the respondent's response vector and the vector of item sample means as a straightforward person-fit index. This index is usually known as the “personal correlation” and will be denoted here by *r*_*pg*_.

As initially proposed, the personal correlation was only intended for binary responses. Because in this case the value a correlation can have heavily depends on the marginal distribution of the data it is difficult to compare values across persons. Furthermore, there is no standard cutoff value for classifying a respondent as inconsistent on the sole basis of the magnitude of his/her personal correlation. Possibly for these reasons *r*_*pg*_ is hardly used nowadays. However, for the approximately continuous item responses considered here, the differential attenuation problem due to marginal differences is considerably minimized. And, regarding the second limitation, *r*_*pg*_ might still have an important role as an auxiliary practical index even when there are no simple cutoff values.

Conceptually *r*_*pg*_ quantifies the similarity between the item locations for the respondent and the normative item locations obtained from the entire group. In other words, *r*_*pg*_ assesses the extent to which the responses of the individual are sensitive to the group-based normative ordering of the items by their extremeness.

#### Model-based indices

We shall propose here a model-based personal correlation index, which we shall denote as *r*_*pm*_, and which is defined as the product-moment correlation between the respondent's response vector (**x**_*i*_) and the vector of expected item scores (${\hat{x}}_{i}$), whose elements are given by

(13) $$\begin{array}{l} {\hat{X}}_{ij}=\mu_{j}+\lambda_{j}{\hat{\theta }}_{i}\left(ML\right)\; \end{array}$$

Conceptually *r*_*pm*_ measures the similarity (in terms of rank ordering) between the scores obtained by the respondent and the scores that would be expected given the structural FA parameters and his/her trait estimate.

#### Relations between residual-based and correlation-based indices

Within each class, MB and GB, the residual and correlational indices are obtained from the same observed-expected vectors and are algebraically related. The basic relations have been discussed above in terms of profile analysis. In this section we shall further analyse the relations in order to show the complementary role that the residual-based and the correlation-based indices can have in practical assessment. We shall focus the analysis on the relations between *r*_*pm*_ and *lico*, which are the most direct ones. The results, however, are still valid in general for both types of index.

By using vector notation and standard covariance algebra, the following result is obtained

(14) $$\begin{array}{l} lico_{i}=\left(\frac{n^{2}}{(n-1)\overset{j}{\underset{n}{\sum }}{\sigma^{2}}_{\varepsilon j}}\right)[{\left(x_{i}-{\bar{\hat{x}}}_{i}\right)}^{2}\\ \;\;\;\;\;\;\;\;\;\;\;+{\left(s(x_{i})-s({\hat{x}}_{i})\right)}^{2}+2s(x_{i})s({\hat{x}}_{i})(1-r_{pm(i)})]. \end{array}$$

The right hand side of Equation (14) separates the elevation (differences in means), scatter (differences in standard deviations), and differences-in-shape components that are measured by *lico*. If the first two components are kept constant, the relation is indeed negative: the higher *r*_*pm*_ is, the lower *lico* Is.

The result (Equation 14) suggests that the effectiveness of the personal correlations and the residual indices will depend on the type of inconsistency. So, if inconsistency mainly affects the rank ordering of the item scores with respect to the group-based normative ordering (*r*_*pg*_) or the model-expected ordering (*r*_*pm*_), then the personal correlations are expected to be more effective than the residual indices. On the other hand, if inconsistency mainly affects the means and variances of the observed-expected vectors, then, residual indices are expected to be more effective. As an example of this second case, consider an extreme respondent who, in everything else, behaves according to the FA model. The expected-observed agreement in terms of rank ordering is perfect in this case. However, the “scatter” and perhaps the “elevation” components differ, because the “high” observed scores are higher than expected while the “low” scores are lower.

We shall finally discuss relations with cutoff values. If the null hypothesis of consistency holds, the expected values of the personal correlations for an individual *i* are found to be:

(15) $$\begin{array}{ll} \begin{array}{l} E\left(r_{pm\left(i\right)}\right)=\sqrt{\frac{\mathrm{var}(\mu_{j})+\theta_{i}^{2}\mathrm{var}(\lambda_{j})}{\mathrm{var}(\mu_{j})+\theta_{i}^{2}\mathrm{var}(\lambda_{j})+{\bar{\sigma }}_{\varepsilon j}^{2}}}\\ \text{ and:}\\ E(r_{pg\left(i\right)})=\sqrt{\frac{\mathrm{var}(\mu_{j})}{\mathrm{var}(\mu_{j})+\theta_{i}^{2}\mathrm{var}(\lambda_{j})+{\bar{\sigma }}_{\varepsilon j}^{2}}} \end{array} & . \end{array}$$

For both *r*_*pg*_ and *r*_*pm*_, the expected value under the null hypothesis of consistency depends on both the item and the person parameters. So, unlike what occurs with *lico* and *licz*, a simple value cannot be rigorously proposed as a cutoff for *r*_*pg*_ and *r*_*pm*_. It is mainly for this reason that we prefer to consider personal correlations as auxiliary indices.

## Simulation studies

### Design and general conditions

We agree with Rupp (2013) that simulation studies should reflect, as far as possible, the inconsistent behaviors that are found in real life, and we have tried to do this here. Because we are mainly concerned with typical-response measurement (i.e., personality and attitude), we have tried to mimic response mechanisms expected to lead to inconsistent responses in this domain (e.g., Ferrando, 2015). We have also tried to provide realistic choices in terms of sample sizes, test lengths, distributions of item/person parameters, and proportion of inconsistent respondents.

The conditions that were kept constant in all the simulations were the following: (a) the item scores were 5-point Likert scored as 1–5; (b) the intercepts μ_*j*_ were randomly and uniformly distributed between 1.5 and 4.5; and (c) the loadings λ_*j*_ were randomly and uniformly distributed between 0.3 and 0.8. As for the rationale of these choices, first, there seems to be agreement that five is the minimum number of categories from which linear FA can be considered to be a reasonable approximation (Ferrando, 2009; Rhemtulla et al., 2012). Second, condition (b) reflects a desirable condition in a general-purpose test: a wide range of difficulties evenly distributed. Finally, conditions (c) and (d) aim to reflect the results we generally find in FA applications in the personality domain.

### Independent variables

The study was based on a 2 × 3 × 3 × 4 × 7 design with the following independent variables: (a) sample size (*N* = 500 and *N* = 1000); (b) test length (*n* = 20, *n* = 40, *n* = 60); (c) percentage of inconsistent respondents (5, 15, 25%); (d) percentage of items in which responses were inconsistent (5, 10, 20, 30%), and (e) type of inconsistent responding. The seven types of simulated inconsistencies are described below.

1. Random responding (RAND). A very common type of misfit (Liu et al., 2016) expected in conditions of unmotivated responding and/or fatigue in the case of long tests. Responses for the corresponding sub-set of items were generated using a random number generator.
2. Low person reliability (LPR) (e.g., Ferrando, 2015). Random responding can be considered as the extreme of a dimension of low person reliability characterized by a certain degree of insensitivity to the normative ordering of the items. This type of inconsistency was simulated here by generating the data according to Ferrando (2014) differential-discrimination model and setting the person parameter to a value of 0.20 for all of the item responses (a unit value is the expected value in the normative model).
3. Sabotaging (SAB). This is the tendency of the respondent to agree with the most extreme or “difficult” items and disagree with the “easier” items (see Ferrando, 2015). For the corresponding sub-set of items, responses at one extreme were changed to responses at the other extreme (e.g., 5–1 or 1–5).
4. Spuriously low unexpected responses (UE-L) and spuriously high unexpected responses (UE-H). There are expected to be inconsistencies of types UE-L and UE-H in some sub-sets of items mainly in the case of multidimensionality, faking (in the subset of socially desirable items), and acquiescence when balanced scales are used (see Ferrando, 2015). For the selected sub-set of items the expected central responses were moved one or two points down (spuriously low) or up (spuriously high).
5. Model-consistent extreme responding (EMC). Extreme responding was considered to be a general source of misfit that affects all items. In type EMC, the direction component of the response (agree-disagree) was model-based but the response was more extreme than expected from the model. This was simulated by moving responses to one or two points above the expected response in the model-expected direction.
6. Partially inconsistent extreme responding (EMIC). First, the simulation proceeded as for type EMC, but then the extreme responses were reversed for 20% of the items. So, for the majority of items the response behavior is model based, but for the remaining items it is “pure extreme responding” regardless of item content.

For RAND, SAB, UE-L, and UE-H, the conditions in (d) above apply. For LPR, EMC, and EMIC, inconsistency was simulated for all of the items, so the common percentage in (d) was 100%.

The general conditions described so far were considered for two scenarios. In the first, the structural (item) parameters μ_*j*_,λ_*j*_, and $\sigma_{\varepsilon j}^{2}$ were assumed to be known from previous calibrations, an “ideal” condition that is commonly used in IRT-based simulations. Although not implausible, this is not the usual situation in FA applications, and its main role here is to provide an upper benchmark for the effectiveness of the MB indices.

The second scenario is the most habitual in FA applications: neither the structural parameters nor the trait levels are known, and they are both estimated from the same sample by using the calibration-scoring procedure described above. Because (a) the item indices are now sample estimates, and (b) the sample contains a certain proportion of inconsistent respondents, the effectiveness of the indices must necessarily be lower than that of scenario 1. In all the conditions here, item calibration was based on Unweighted Least Squares (ULS) estimation for two reasons. First, ULS is quite robust and can be used with small-to-medium samples and relatively large models (Jöreskog, 2003), the most common situation in typical-response applications. Second, when the model to be fitted is not exactly correct but only an approximation (as discussed above), ULS tends to produce more accurate estimates than other theoretically superior procedures (e.g., Briggs and MacCallum, 2003).

Overall, the general design so far summarized had 684 different conditions. The number of replications per cell was 500.

### Assessing the effectiveness of the indices

Effectiveness of a person-fit index can be defined as its ability to reliably detect disturbances of various types (e.g., Karabatsos, 2003). In this study, we are particularly interested in the seven types of disturbances described above. We used two approaches to assess effectiveness: the first studied the mean differences in the consistent and inconsistent groups, and the second, more graphical approach was based on Receiver Operating Curve (ROC) analysis.

In the first approach we used, Hedges's *g* effect size index as a simple summary measure. It was calculated for all the person-fit values and design cells. This index provides a general idea about the potential capability of the index for differentiating consistent and inconsistent respondents in an easily interpretable metric.

In the second approach, ROC curves were estimated and graphically displayed so that each graph showed (a) the curves corresponding to the five indices compared, (b) the diagonal line of no differentiation, and (c) the optimal operating point (defined below). As a summary of the ROC analysis we computed (a) the estimated area under the curve (AUC), and (b) the optimal operating point (OOP), which was estimated by using an un-informative prior. The first measure provides an overall summary of the index effectiveness. The second is of interest for suggesting plausible cutoff values. The ROC analysis was performed with the MATLAB Toolbox *perfcurve* routine (available at https://es.mathworks.com/help/stats/perfcurve.html).

## Results

### General results

In both scenarios, Table 1 shows the overall results for the mean-comparison approach across all the conditions in the study. The table clearly reveals some general trends. As far as the MB indices are concerned, the means and standard deviations of *lico* and *licz* in the consistent groups (i.e., when the null hypothesis holds) are reasonably close to their expected values. It is also clear that, as expected, the effectiveness of the MB indices is substantially higher in scenario 1, and is especially high for *r*_*pm*_ and *lico*. In scenario 2, however, *lico* is the most effective MB index, and its effectiveness still seems to be good in this more realistic scenario.

**Table 1:** **Mean-group comparisons: general results**.

		**GB**		**MB known parameters**			**MB sample calibration**		
		**Bollen's a_ii_**	***r*_pg_**	**lico_i_**	**licz_i_**	***r*_pm_**	**lico_i_**	**licz_i_**	***r*_pm_**
Inconsistent responses	$\bar{X}$	0.066	0.546	2.414	2.840	0.493	1.397	1.075	0.664
	S_x_	0.042	0.260	1.720	3.610	0.329	0.614	1.187	0.185
Consistent responses	$\bar{X}$	0.051	0.756	1.001	0.120	0.778	0.940	−0.088	0.774
	S_x_	0.032	0.075	0.268	1.013	0.070	0.268	0.890	0.071
Effect size (g)		0.447	1.77	1.99	1.62	1.99	1.35	1.25	1.17

We turn now to GB indices, which are only displayed once in Table 1 because they do not depend on the model parameters. First, Bollen's *aii* is the least effective index, which was also expected because it was not designed specifically to detect inconsistent patterns but outliers in general. In contrast, *r*_*pg*_ shows a high amount of effectiveness and is the index that performs best when the structural parameters have to be estimated from the sample.

**Figure 2:**
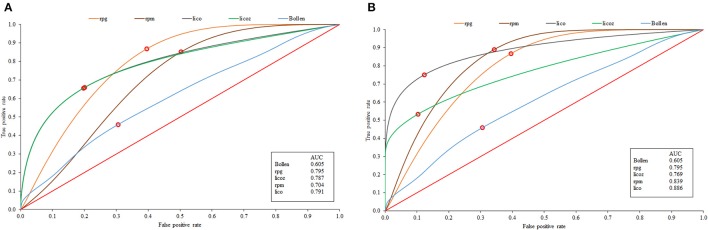
**ROC curves corresponding to the generals results for the known-parameter scenario (A)**, and sampled-calibrated items **(B)**.

The ROC results for the mean-comparison results discussed so far are in Figure 2. Figure 2A shows the results for scenario 1 and Figure 2B for scenario 2. The results are in close agreement with those in Table 1 (the correlation between effect size and the AUC is 0.94). Note that Bollen's index is not far from the diagonal line of no differentiation, and *r*_*pg*_ is the furthest from it. Note also that in Figure 2A *lico* and *licz* completely overlap, whereas in Figure 2B *lico* appears to be more effective than its standardized version, and overall is again the most effective MB index when items are sample-calibrated.

### Specific results

The results of the simulation are too numerous to be discussed here in detail. So, we shall provide only a summary of the most important of them. Full results are available from the authors.

We start with the non-significant results. There were no noticeable differences regarding sample size for any of the indices, possibly because a sample of *N* = 500 is large enough to provide stable results.

**Figure 3:**
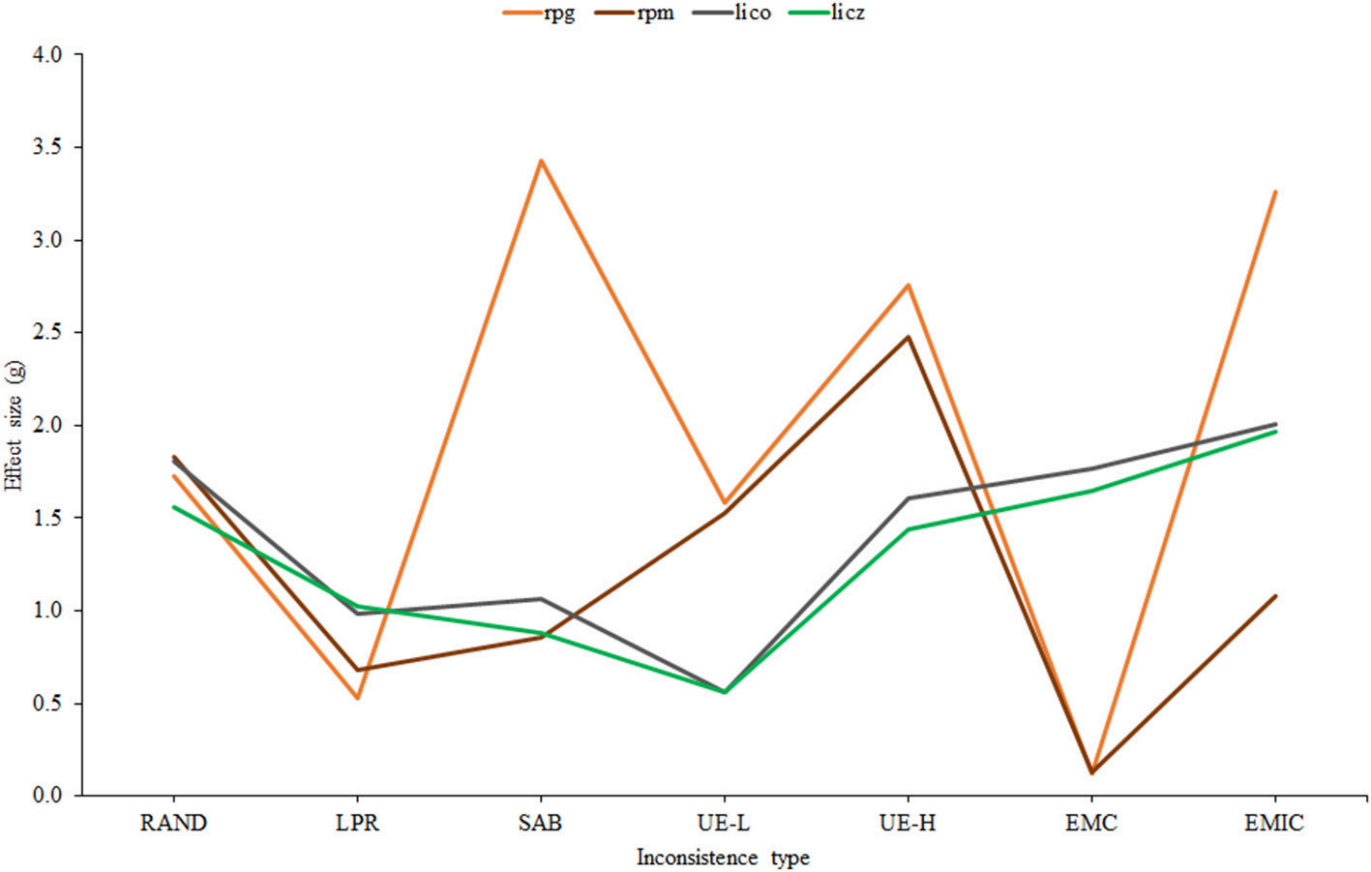
**Effect size estimates corresponding to the selected indices across different types of inconsistency**.

Figure 3 shows the effect-size estimates of effectiveness plotted against the seven different types of inconsistency. For clarity, Bollen's *a*_*ii*_ has been omitted, and only the sample-calibrated results are presented for *lico, licz*, and *r*_*pm*_.

First, as expected, *lico* and *licz* have very similar profiles. However, as suggested by Table 1, Figure 2, the effectiveness of *lico* appears to be slightly but consistently higher than its standardized version. Second, the profiles of the correlational indices are similar one to another except for the fact that the simple *r*_*pg*_ considerably outperforms *r*_*pm*_ in EMIC and SAB. Finally, as also found in Table 1, Figure 2, *r*_*pg*_ is the most effective index overall (when item parameters are not known). However, it is not consistently superior, and *lico* appears to be more effective in LPR and, above all, EMC, as was predicted above. Taking into account all the results so far, a reasonable choice for practical applications would be a combination of *lico* and *r*_*pg*_. And these are the only indices that we shall consider from now on.

**Figure 4:**
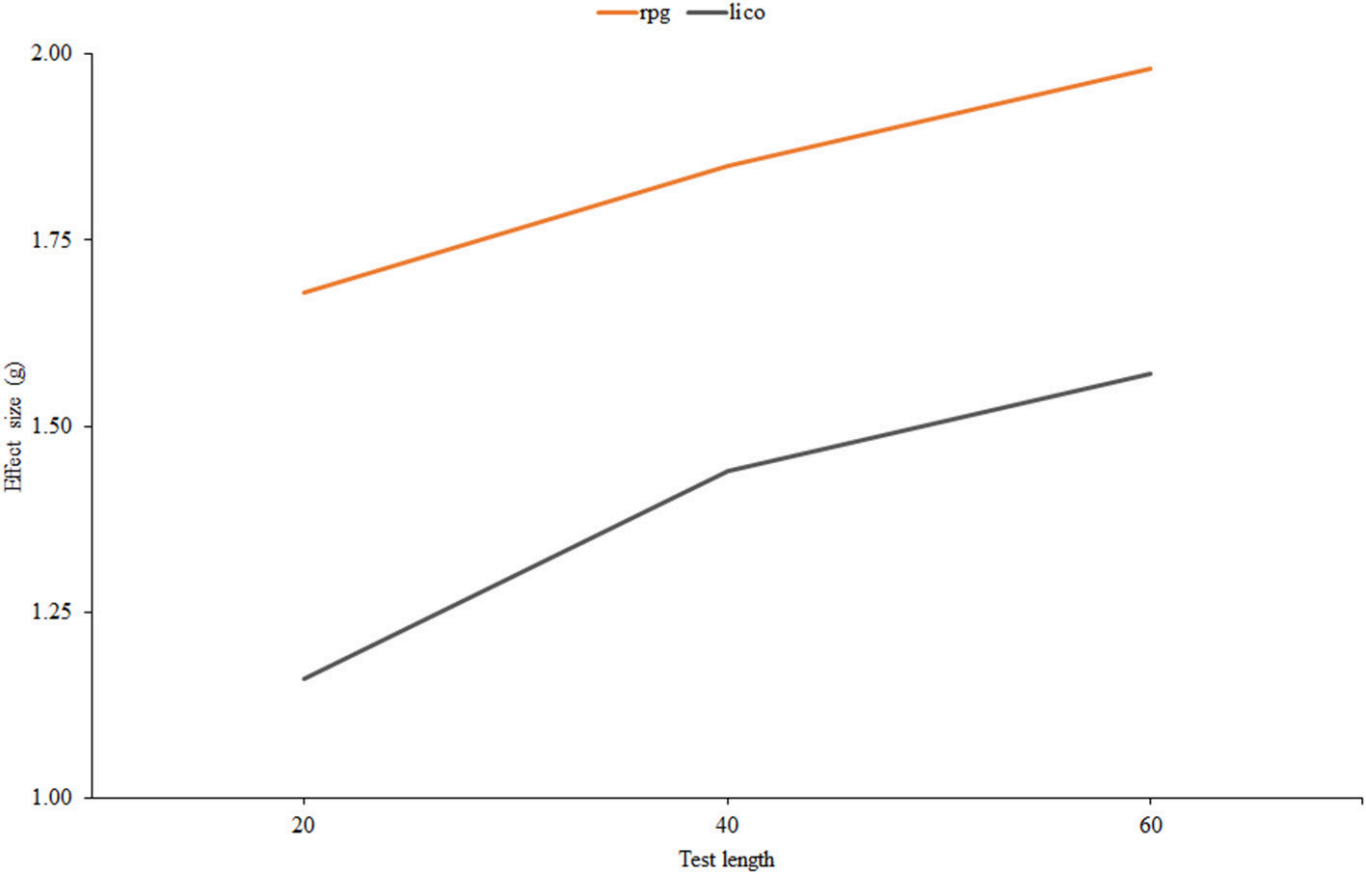
**Effect size estimates for ***r***_***pg***_ and ***lico*** as related to test length**.

For the two indices selected, Figure 4 shows the effect-size estimates of effectiveness plotted against test length. In both cases, effectiveness increases with the number of items. It is generally higher for *r*_*pg*_ but there tends to be fewer differences between them as the test becomes longer, and, furthermore, these differences are rather small in AUC units. In the 60-item condition, the results in Figure 4 correspond to an AUC of 0.82 for both indices, which means a respectable amount of effectiveness. At the other extreme, for 20 items the AUCs would be of 0.74 for *lico* and 0.76 for *r*_*pg*_, which are relatively low. Overall then, the results are similar for both indices, and agree with what has been reported in the person-fit literature: practical indices are generally ineffective in short tests of fewer than 20 items, and effectiveness increases mostly as a function of test length (e.g., Ferrando, 2015).

**Figure 5:**
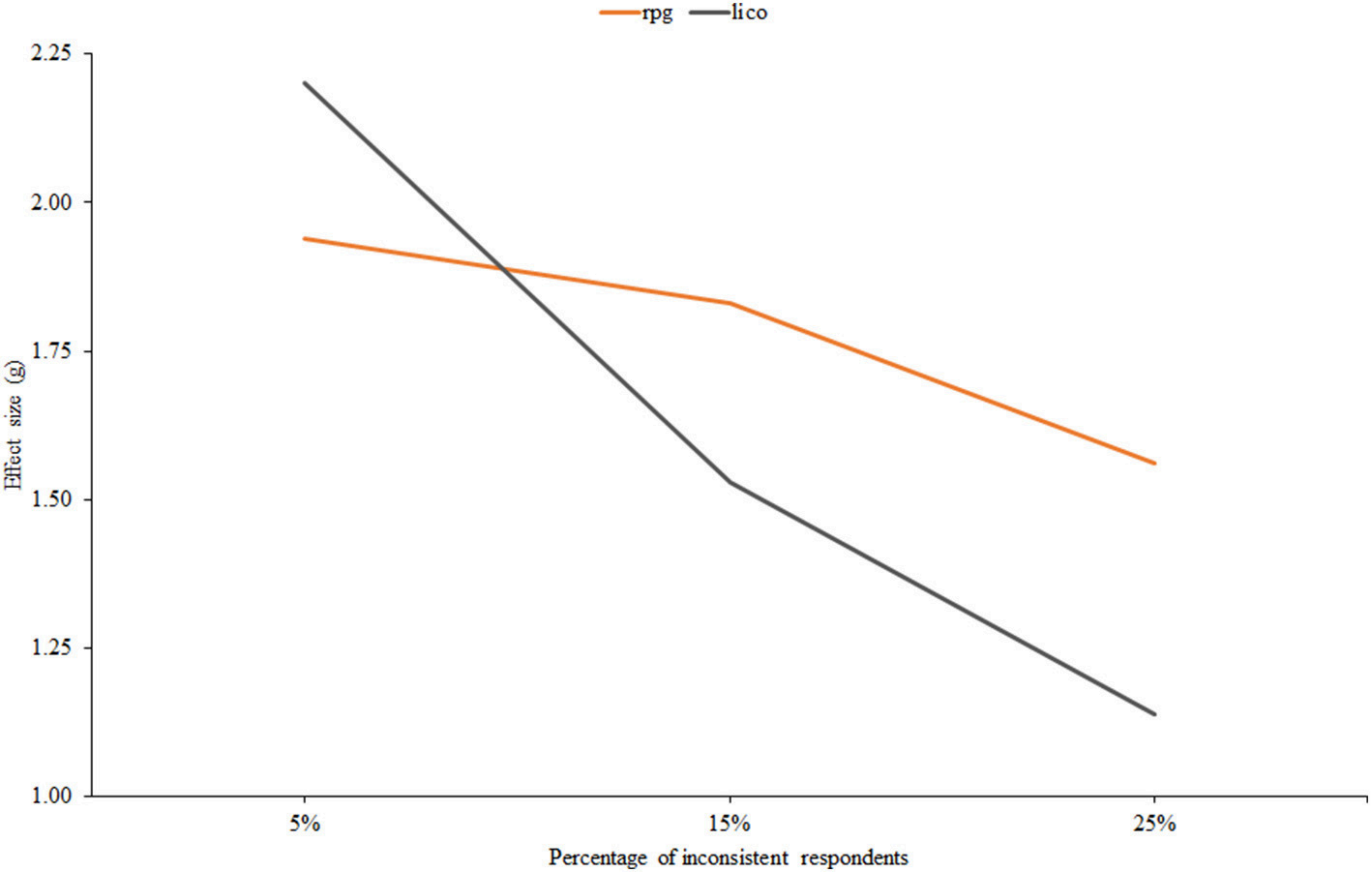
**Effect size estimates for ***r***_***pg***_ and ***lico*** as related to the percentage of inconsistent respondents**.

Figure 5 displays effect size against the percentage of inconsistent respondents, and results are again in accordance with the person-fit literature: effectiveness decreases as the proportion of inconsistent individuals increases. Note also that the decrease is more pronounced for *lico*, and that this index would be expected to be more effective than *r*_*pg*_ when the proportion of inconsistent respondents is low: at the 5% level, the AUC of *lico* is 0.90 against a 0.82 value for *r*_*pg*_.

**Figure 6:**
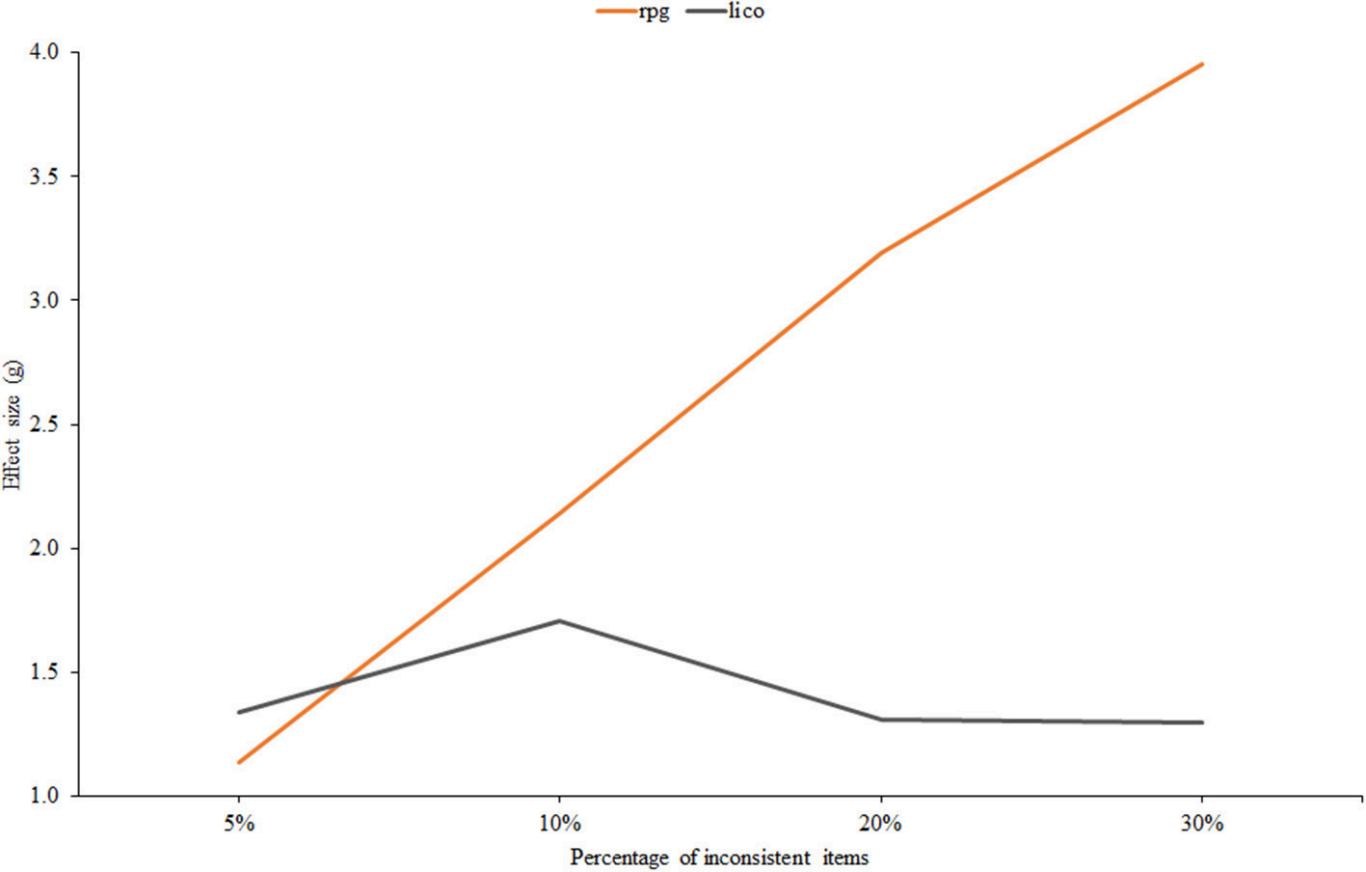
**Effect size estimates for ***r***_***pg***_ and ***lico*** as related to the percentage of inconsistent items**.

Finally, Figure 6 displays effect size against the percentage of items in which inconsistent responses were given. It is in this condition that the two indices differ most. The effectiveness of *r*_*pg*_ clearly increases with the proportion of inconsistent items while the effectiveness of *lico* tends to decrease. Furthermore, at the 30% level the difference in terms of AUC is considerable: 0.76 for *lico* against 0.98 for *r*_*pg*_. The most plausible explanation for this divergent behavior is the MB vs. GB nature of both indices: as the proportion of inconsistent items increases, the item parameter estimates at the calibration stage become more and more degraded, and this, in turn, decreases the effectiveness of the person-fit index via the mechanism explained below. In ‘ort of this explanation, we note that, when the item parameters are known (scenario 1), the trends of *lico* and *r*_*pg*_ in this condition are the same.

### Cutoff values

For the sample-based *lico*, Table 2 shows the empirical standard deviations, the approximate expected standard deviations given by *sqrt(2/(n-1))*, and the cutoff values obtained from (a) the OOPs, and (b) Equation (11).

**Table 2:** **Standard deviation and cutoff values for ***lico*** as related to test length**.

**Num. items**	**Sd-Known parameters**	**Sd-sample parameters**	**Expected Sd**	**OOP Known parameters**	**OOP-sample calibration**	**$1+\frac{2}{\sqrt{n}}$**
20	0.346	0.336	0.32	1.381	1.141	1.447
40	0.238	0.224	0.23	1.292	1.129	1.316
60	0.197	0.210	0.18	1.279	1.121	1.258

The results in Table 2 are interesting. First, the empirical standard deviations decrease with test length, just as it should be, and agree rather well with their expected values. Second, the simple cutoff values in Equation (11) proposed by Smith et al. (1998) are quite close to the OOPs when the item parameters are taken as known. For the sample-calibration case, however, cutoff values determined by 1+ *sqrt(2/(n-1)* (i.e., expected mean plus one expected standard deviation) will be closer to the corresponding OOPs. To sum up, it appears that simple cutoff values that only depend on test length can be proposed for practical applications based on *lico*. And further, the conventional 1.3–1.5 values proposed in Rasch modeling as a plausible general cutoff would possibly work reasonably well in practice.

## Illustrative example

The short example provided in this section uses empirical data collected in personality research, and aims to (a) illustrate how the proposal made in the article can be used in practical applications, and (b) obtain further information regarding the behavior of the two chosen indices in real datasets when the conditions for effective person-fit assessment are far from ideal.

An 18-item Spanish version of Ray's balanced dogmatism scale (BDS, see Ferrando et al., 2016) was administered to a group of 346 undergraduate students. The items of this scale used a 6-point Likert format ranging from “completely disagree” (1) to completely agree (6).

First, the unidimensional FA model was fitted to the data using robust ULS estimation as implemented in version 10.4 of the FACTOR program (Lorenzo-Seva and Ferrando, 2013). The fit of the model was reasonably good (details can be obtained from the authors). Next the structural parameter estimates (μ,λ, and σ^2^) were taken as fixed and known values, and (a) the ML trait estimates and (b) the two indices proposed in this article were obtained using the new procedures implemented in FACTOR.

Inspection of the BDS item scores revealed that the items of the scale were “medium” to “easy,” with means ranging from 3.08 to 5.73 (recall that the possible range of scores is 1–6). The lack of a wider range of item difficulties clearly diminishes the effectiveness of any person-fit measure (Ferrando, 2015), but is expected to have particular impact on the functioning of *r*_*pg*_ (see Equation 15). As the variability of the vector of item means decreases, the expected *r*_*pg*_ value approaches zero and the estimate becomes more unstable. This prediction was supported by the results: the mean value of *r*_*pg*_ in the sample was 0.53, lower than the usual values obtained in the simulation. The correlation between *r*_*pg*_ and *lico* was −0.41, which goes in the expected direction (see Equation 14) and indicates a moderate degree of agreement between both measures that would have been expected to be higher if the range of item difficulties had been wider. Finally, *r*_*pg*_ was obtained for all the respondents, which means that no single-category respondents appeared in the data. Overall, and in spite of the less than ideal conditions *r*_*pg*_ is still expected to be useful here as an auxiliary index.

**Figure 7:**
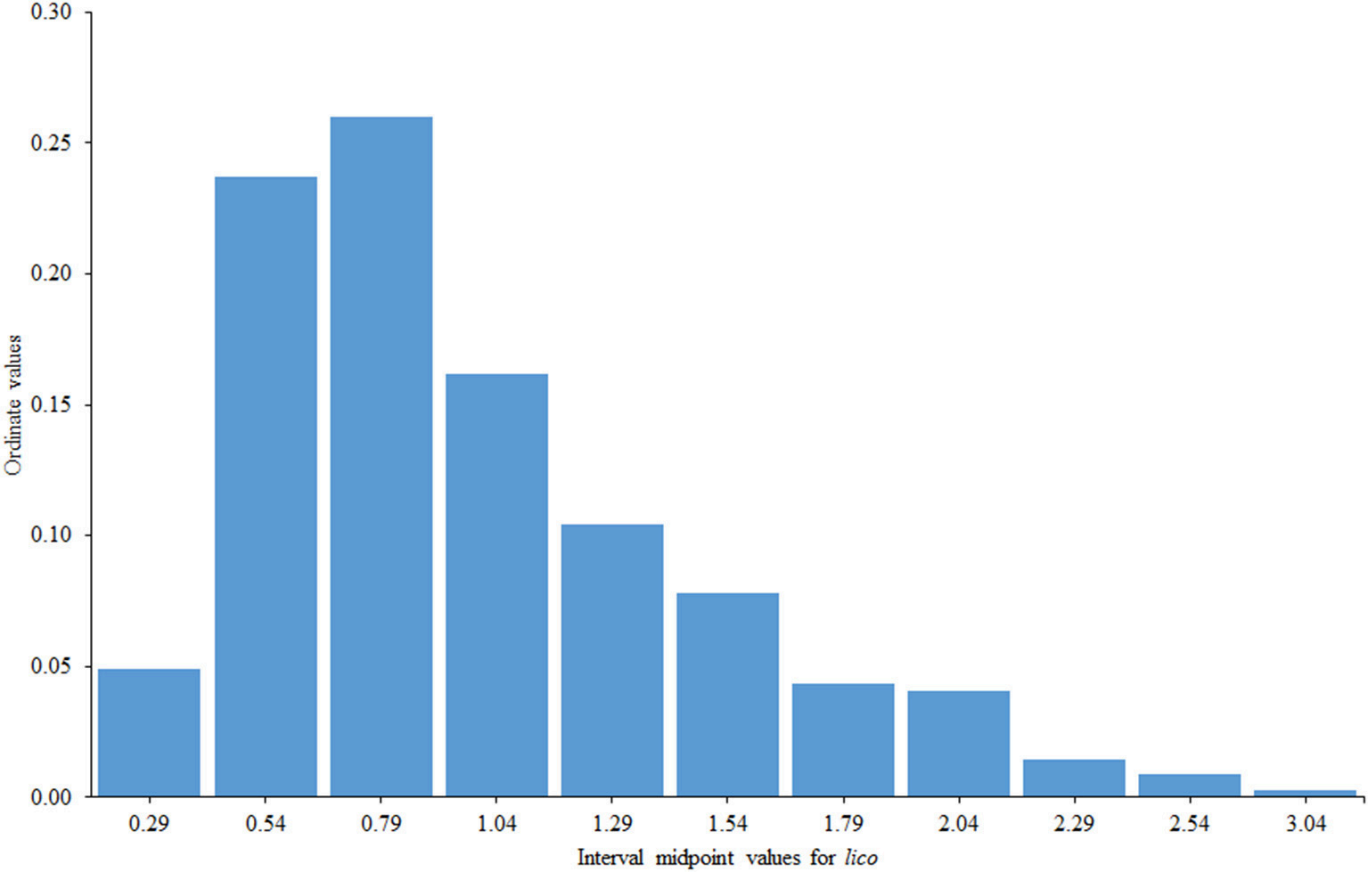
**Distribution of ***lico*** values in the illustrative example**.

*Lico* seemed to work rather well even in these relatively unfavorable conditions (short test with a reduced range of item difficulties). The mean value of *lico* was 0.99 (virtually its expected value) and the corresponding standard deviation was 0.49, which is somewhat above the expected value of 0.34 (approximate) for *sqrt(2/(n-1))*. This result is only to be expected if the sample contains a certain proportion of inconsistent respondents. The distribution of the *lico* values can be seen in Figure 7.

The right tail of the distribution in Figure 1 presumably contains those subjects who responded inconsistently with the FA model and with whom we wish to identify. We used Smith's critical value in Equation (11) (i.e., 1.47 with 11 items) and flagged 55 respondents (16% of the sample) as potentially inconsistent. Inspection of the corresponding patterns using the procedures proposed by Ferrando and Lorenzo-Seva (2016) suggested that the main sources of inconsistency were: (a) model-based extreme responding (characterized by a high value of *lico* and an above-average value of *r*_*pg*_), (b) unexpected responses to certain sub-sets of items (in this case *r*_*pg*_ was generally low), and, to a lesser extent, (c) random responding/low person reliability (characterized by a near zero value of *r*_*pg*_). Two possible cases of sabotaging or malingering (characterized by a high value of *lico* and a strong negative *r*_*pg*_value) were also identified.

## Summary, proposal and implementation

The results described so far suggest that the combined use of *lico* and *r*_*pg*_ would be an effective first-step approach for flagging potentially inconsistent respondents in applications based on the standard FA model. The indices selected show a different profile of effectiveness across different types of inconsistency (Figure 3), and they also behave differently in terms of the proportion of items which are answered inconsistently (Figure 6). As for similarities, both essentially depend on the general conditions that affect person-fit indices (Ferrando, 2015): their effectiveness mostly depends on test length and decreases as the proportion of inconsistent respondents increases. Furthermore, the results of the empirical example show that a reduced range of item difficulties diminishes the effectiveness of the indices, especially that of *r*_*pg*_. They also show, however, that even in the case of a relative short test with a reduced range of difficulty, the proposed indices work reasonably well. Overall, we believe that in a test with a minimal length of about 25 items and in which the proportion of inconsistent respondents is relatively small (say <10%), the approach proposed here would be expected to be highly effective in practice.

As discussed below, we do not feel that the present results allow strict cutoff values to be proposed for the selected indices. The expected values of *r*_*pg*_ depend on too many factors, so it seems better to use it as an auxiliary index, as proposed. As for *lico*, the cutoff values in Equation (11) seem to work reasonably well, but the results of the illustrative study suggest that they might even be too sensitive (16% of inconsistent respondents in a sample of volunteers seems to be a bit too high).

Because the results of the study are encouraging and a workable proposal can be made, the indices chosen and the reference values discussed above have been implemented in version 10.4 of the program FACTOR (Lorenzo-Seva and Ferrando, 2013), a free, comprehensive program for fitting the FA model. Furthermore, Matlab functions and illustrative data are offered as Supplementary Material.

## Discussion

Simple and effective practical indices based on the linear FA model can be used and easily implemented (as they have been) in a standard FA program. These are the main conclusions of the present study. At the same time, however, the study does have some limitations, and the results point out issues that can be improved or that deserve further research.

We shall start with a caveat. Practical person-fit indices are non-specific screening devices for tracing potentially inconsistent respondents. Ideally, however, once a pattern has been flagged as potentially inconsistent, further information should be obtained regarding (among other things) (a) the type of inconsistency, and (b) the impact that the inconsistency has on the trait estimates (Emons et al., 2005). FA-based analytical and graphical procedures for obtaining this information already exist and are implemented in stand-alone programs (Ferrando and Lorenzo-Seva, 2016). The problem may be how to link the first-step results obtained with a general FA program to this second-step type of analysis.

An alternative approach to using a first-step practical index followed by a second-step *post-hoc* analysis is to include the expected sources of misfit directly in the model (if this information is available). As far as we know, to date proposals of this type intended for the FA framework have been made for three sources of misfit: person unreliability (Ferrando, 2011), model-based extreme responding (Ferrando, 2014), and acquiescent responding (Ferrando et al., 2016). In this alternative approach, the use of the practical indices we propose has a secondary but important role that deserves further research: to detect the remaining inconsistent response patterns once the main expected sources of misfit have been explicitly taken into account in the model.

We turn now to more specific limitations and potential improvements. One clear limitation is that the study is only concerned with unidimensional FA solutions. In principle, our proposal is expected to work well not only with essentially unidimensional measures, but also with multidimensional instruments analyzed on a scale-by-scale basis, and instruments that behave according to a dominant factor solution (e.g., those that can be fitted with a bi-factor solution (see Reise, 2012). Even so, we acknowledge that many typical-response instruments are truly multidimensional questionnaires.

Since the personal correlation *r*_*pg*_ is a GB index, it can be obtained with no need to fit the FA model, and so it can be applied directly regardless of the number of factors. As for *lico*, its multidimensional extension is straightforward. So, the problem is not whether the indices generalize to the multidimensional case, but rather whether in this case they will be as effective as in the unidimensional setting. This point clearly requires further research.

The effectiveness of *lico* decreases when the trait estimates are poor (unreliable and/or indeterminate) and when the item parameters have to be estimated from the sample. These are important limitations. As for the first issue, we recommend checking the general quality of the trait estimates first by using marginal reliability measures and measures of factor indeterminacy such as Guttman's index (Guttman, 1955), before starting person-fit analysis. As for the second problem, Nering (1997) suggested one possible solution based on a two-stage calibration process in which (a) initial calibrations were run to identify potentially inconsistent patterns, (b) these patterns were removed from the data, and (c) items were recalibrated in the “cleaned” sample. It will be worth trying procedures of this type to see if levels of effectiveness can be obtained that are close to those achieved in the known-parameters scenario.

Finally, further research on cutoff values could be of interest. The simple cutoff criteria in Equation (11) considered here appear to work reasonably well as a starting point, but further study is required, and future substantive applications could also help to refine the proposal. On the other hand, person-based cutoff values obtained for each pattern using simulation (van Krimpen-Stoop and Meijer, 1999) could be a better alternative. Although they do require additional intensive computation, they are otherwise easily implemented.

In spite of the limitations discussed so far, we believe that what we propose here is a useful tool that allows the practitioner to routinely assess person fit in FA-based psychometric applications. As discussed above, this type of assessment is of considerable importance, so we hope that our proposal will be widely used in the near future.

## Author contributions

PF initiated the paper, advised on simulation conditions and the choice of indices tested, and coordinated team meetings. AV proofread and provided recommendations. UL conducted the simulation studies and summarized the outcomes.

## Funding

The research was supported by a grant from the Catalan Ministry of Universities, Research and the Information Society (2014 SGR 73), and by a grant from the Spanish Ministry of Education and Science (PSI2014-52884-P).

### Conflict of interest statement

The authors declare that the research was conducted in the absence of any commercial or financial relationships that could be construed as a potential conflict of interest.

## Supplementary material

The MATLAB functions to compute the selected indices (*lico* and *r*_*pg*_) as well as the data corresponding to the illustrative example are made available to the interested researchers.
