# Supplementary material for: Practical Person-Fit Assessment with the Linear FA Model: New Developments and a Comparative Study
Source: Front Psychol. 2016 Dec 27;7:1973. doi: 10.3389/fpsyg.2016.01973 (PMC5186803; doi:10.3389/fpsyg.2016.01973)
Supplement: Supplementary file 1 [file DataSheet1.DOC]

ILLUSTRATIVE EXAMPLE DATA

PARTICIPANTS' RESPONSES

Participants: N=346

Items: 18

Respostes:

5 6 5 6 6 6 5 5 6 6 5 2 4 6 6 6 6 6

4 1 5 5 5 6 6 2 2 6 5 2 2 6 5 5 6 4

2 6 1 3 4 5 6 6 5 6 2 3 5 6 5 6 1 6

4 5 3 4 5 6 6 3 2 6 5 3 4 6 3 6 4 4

5 6 4 5 5 4 5 6 4 6 5 4 4 6 5 5 5 6

4 6 3 6 4 5 6 1 4 6 6 4 6 6 3 6 6 6

5 5 3 5 5 5 5 3 4 6 3 6 6 6 5 5 6 6

6 6 1 6 5 5 6 3 5 6 4 3 4 6 6 6 4 5

4 6 3 5 4 6 5 2 5 6 4 4 5 6 3 6 2 4

5 6 4 5 6 6 6 3 5 6 4 4 5 6 5 5 4 6

4 5 3 4 5 5 3 4 4 6 5 2 3 5 4 5 2 3

6 6 5 5 5 5 5 2 6 6 5 5 6 6 5 6 5 3

4 6 4 5 6 6 6 1 6 6 6 4 5 6 6 6 2 6

5 5 5 4 5 4 5 4 5 6 5 3 5 5 5 5 5 2

5 6 4 6 6 5 5 2 3 6 6 2 6 6 5 6 3 6

5 5 2 5 4 5 5 2 4 5 5 4 5 3 6 5 3 5

5 6 5 5 2 6 6 5 2 6 4 2 2 5 6 5 6 2

6 6 1 3 6 5 6 6 4 6 1 5 6 6 6 6 6 6

6 6 5 4 4 5 6 3 5 6 6 1 3 6 4 6 5 6

6 4 3 5 5 5 5 5 6 5 4 5 4 5 5 6 3 4

5 5 3 3 6 5 5 3 5 6 4 3 4 6 3 5 4 4

6 6 4 6 5 4 6 1 6 6 1 2 5 6 5 6 3 5

4 4 1 3 4 4 5 1 4 6 4 3 4 2 5 3 5 6

5 4 4 4 4 4 5 4 6 6 5 4 5 3 5 5 4 3

4 6 3 3 4 5 6 2 4 6 3 3 5 5 6 6 5 5

6 6 3 6 5 5 6 2 6 6 5 4 3 6 5 4 4 4

5 5 5 6 5 6 6 1 6 6 6 3 4 6 3 3 5 6

5 5 1 5 6 6 6 4 5 6 5 4 3 5 3 5 6 5

6 5 4 4 5 5 4 5 4 6 5 4 4 5 4 6 3 4

5 5 1 3 3 5 6 4 5 5 4 3 3 6 4 4 5 5

4 6 1 6 4 6 5 6 6 6 6 6 5 6 6 4 2 6

4 6 5 6 6 5 6 5 6 6 6 3 4 4 3 5 4 3

5 6 2 4 3 4 6 4 5 6 4 4 5 5 6 5 4 5

5 6 3 6 6 6 6 2 6 6 5 2 5 6 6 6 6 3

6 6 4 2 2 5 6 3 4 6 6 6 6 6 6 6 5 6

6 5 6 6 6 5 4 5 5 6 4 5 5 6 5 4 5 4

4 6 3 4 2 5 6 2 6 6 5 4 2 5 4 5 6 4

2 6 2 1 2 5 5 1 3 6 6 1 2 6 4 4 6 4

4 5 5 6 6 4 6 5 6 6 5 5 5 6 4 6 4 4

5 6 4 4 5 5 6 1 6 6 5 6 5 6 1 6 6 5

5 5 2 2 3 4 5 2 2 5 6 2 1 5 3 5 4 2

6 6 2 5 5 5 5 4 6 6 5 3 3 6 5 5 6 2

3 6 2 6 4 5 6 4 4 6 3 6 2 6 5 6 6 5

4 6 3 6 6 5 6 3 6 6 6 6 5 4 6 6 5 5

5 5 5 4 4 4 5 1 5 6 5 3 5 4 2 5 4 2

5 5 4 3 4 5 5 1 4 6 5 3 4 5 4 5 6 5

3 5 4 5 5 5 5 2 5 6 4 2 5 6 3 6 5 3

4 2 3 6 5 6 6 3 3 6 6 6 5 6 6 4 6 5

6 6 5 6 5 6 6 6 6 6 6 3 4 5 5 3 4 4

6 6 1 4 6 6 5 3 3 6 6 6 2 5 3 6 6 6

5 6 2 3 3 4 5 4 3 6 6 3 4 6 4 5 3 5

4 6 3 6 6 6 5 3 5 4 3 4 6 5 6 6 6 6

5 5 1 3 3 5 4 3 4 6 4 5 5 6 4 5 6 3

6 6 5 2 1 6 6 2 3 6 5 1 2 6 3 6 2 6

6 6 6 6 4 6 6 6 6 6 5 6 6 6 5 6 5 6

6 5 4 5 2 5 6 1 2 6 6 1 6 4 6 5 5 6

4 5 4 6 6 6 5 6 6 6 6 6 5 6 6 5 5 6

3 4 2 4 4 4 5 1 3 6 5 4 3 6 4 5 6 3

6 6 3 3 4 6 5 1 5 6 3 4 3 6 4 6 6 4

5 5 2 4 5 6 5 2 2 6 3 3 5 6 2 2 6 2

4 4 3 3 5 4 6 2 5 6 4 3 4 5 4 6 3 3

2 2 2 1 1 1 6 1 3 6 6 6 1 1 1 4 2 1

4 5 3 2 5 6 5 2 5 6 3 2 3 6 5 5 4 4

3 5 4 3 5 5 5 4 4 6 2 4 3 6 4 4 6 3

5 5 5 4 4 5 5 1 3 6 4 5 5 3 4 1 3 2

5 6 1 5 2 4 4 3 6 6 5 3 3 6 3 4 6 1

6 6 3 3 6 6 6 2 4 6 6 5 3 5 6 6 6 3

4 5 3 4 4 4 4 3 6 5 4 4 4 4 4 4 5 5

5 6 2 6 5 6 6 3 6 6 6 6 4 6 6 5 4 2

5 6 6 2 6 6 5 3 5 6 3 3 4 6 4 4 6 3

6 4 2 5 5 5 5 4 5 6 5 1 5 6 6 5 3 2

2 5 4 6 5 5 4 5 6 6 4 3 5 5 5 5 4 6

5 4 5 4 5 6 6 2 5 6 5 5 5 5 6 5 6 6

3 6 3 3 4 5 5 2 4 6 4 2 5 6 3 3 6 4

5 5 2 5 6 5 6 4 5 6 3 3 4 6 5 4 4 4

5 5 5 3 6 6 6 2 5 6 6 3 2 6 6 4 3 5

5 6 3 4 6 6 5 6 5 6 3 4 5 5 5 6 6 3

5 5 1 5 5 6 6 3 2 6 5 4 6 5 6 5 5 4

4 5 3 5 2 5 5 3 5 6 5 4 6 6 4 5 5 5

5 6 2 2 5 6 6 2 4 6 6 4 3 6 3 4 5 3

6 6 4 4 5 5 5 6 5 6 5 5 4 6 5 6 3 5

5 6 3 3 4 6 6 1 5 6 5 3 3 5 5 3 5 4

4 5 6 4 5 5 6 6 6 6 3 4 5 6 5 5 5 5

6 6 1 5 5 5 5 3 3 6 2 5 5 6 5 6 6 4

5 6 1 5 5 2 6 4 4 6 6 5 3 5 4 4 3 3

5 6 4 5 6 5 5 3 6 6 5 4 5 6 2 5 3 6

5 5 3 2 2 6 6 4 5 6 5 5 4 6 5 5 6 2

4 6 4 3 5 5 6 5 4 6 1 3 4 6 6 6 4 4

5 6 2 2 2 3 5 5 5 6 2 6 4 5 5 6 5 6

4 6 2 5 4 5 6 1 5 6 5 6 5 4 5 4 6 5

4 5 4 4 5 5 5 1 4 5 3 3 4 6 5 5 6 6

6 5 2 4 2 4 5 2 3 6 6 3 3 3 2 5 4 4

3 6 3 5 5 6 6 2 5 5 6 4 4 4 5 3 2 4

4 4 3 5 5 4 5 3 4 6 4 4 4 6 5 4 5 5

4 5 5 5 2 3 6 1 6 6 6 1 2 6 4 1 6 5

6 6 2 5 6 6 5 3 5 6 4 4 2 6 4 6 6 5

6 5 2 5 6 5 5 3 5 6 1 5 5 6 5 6 5 4

4 5 4 5 5 5 5 3 4 6 4 5 3 2 4 5 5 5

6 5 2 3 6 6 6 2 3 6 5 5 5 6 4 4 6 6

6 5 3 5 4 4 5 2 4 6 4 3 3 6 4 6 5 5

5 6 3 3 5 6 3 3 6 6 5 3 5 5 6 4 5 6

3 5 2 4 5 4 3 2 5 6 4 4 4 4 6 3 3 6

4 5 5 2 5 5 4 3 3 5 4 3 5 6 4 4 5 4

5 5 2 5 5 6 5 1 4 6 4 4 5 6 3 6 5 4

6 5 6 4 5 6 4 4 2 6 4 3 5 5 5 3 6 6

5 5 3 3 5 3 4 5 2 3 4 2 4 5 2 2 4 2

6 5 3 4 6 5 6 4 4 6 6 5 3 4 4 5 5 5

4 4 2 5 6 5 5 6 6 5 4 4 5 3 5 3 2 5

6 5 4 6 6 6 6 5 5 5 5 6 3 5 4 6 4 6

4 6 2 6 5 3 6 6 6 6 5 4 5 6 4 6 6 4

4 5 5 6 2 4 6 6 6 6 3 6 6 1 3 6 4 6

6 6 3 5 5 5 6 6 6 6 4 1 3 3 6 6 6 5

5 3 4 3 4 5 5 3 4 6 4 3 3 6 5 4 5 3

2 5 6 5 6 6 6 4 5 6 3 5 6 6 6 5 6 5

3 5 4 4 3 5 4 3 5 5 5 4 4 4 3 4 3 3

4 5 4 3 5 6 6 2 5 6 5 6 6 6 5 4 6 6

3 6 1 3 1 4 3 3 6 5 6 6 4 4 3 3 6 3

6 6 2 6 4 2 5 6 3 5 6 6 5 5 4 4 4 5

4 5 4 5 6 4 3 4 4 5 4 3 3 6 3 5 5 4

5 6 3 4 5 6 1 4 4 6 5 1 1 6 4 6 6 5

4 4 4 4 6 4 6 1 4 6 4 4 4 6 5 5 3 3

5 5 4 6 6 5 6 1 6 6 6 4 3 6 6 6 2 6

5 6 1 1 5 5 5 5 4 4 3 5 5 6 2 5 5 2

5 6 4 6 5 4 1 3 6 6 2 6 5 6 6 6 3 4

6 5 5 6 6 4 6 6 5 6 5 6 6 5 6 5 5 6

6 6 5 6 5 5 5 4 2 6 3 5 5 6 3 4 6 6

4 5 6 5 6 6 6 5 6 6 3 5 6 6 6 6 6 6

5 5 2 6 5 6 6 4 4 6 5 3 6 5 5 4 5 4

4 5 4 4 3 4 4 1 4 6 3 3 3 6 5 4 4 4

6 5 3 4 6 3 4 2 6 4 5 4 4 6 5 4 3 2

4 6 4 5 6 5 6 4 4 6 5 4 3 6 6 5 2 4

5 6 2 6 5 4 5 3 5 6 5 3 5 6 6 6 2 6

3 6 3 6 6 5 6 4 2 6 5 3 4 6 5 5 5 4

4 5 2 4 4 5 5 3 6 6 5 5 5 6 5 5 5 3

4 5 2 4 4 3 6 2 4 5 4 4 3 6 3 5 2 3

5 6 3 5 4 5 6 3 6 4 5 2 6 6 4 6 3 5

2 5 3 5 6 4 5 3 4 5 3 6 4 6 3 4 3 5

5 6 1 5 6 6 6 4 6 6 5 5 6 6 6 6 3 5

2 5 4 1 4 5 6 5 6 6 4 5 6 6 5 6 6 6

5 5 4 3 4 4 5 3 4 6 4 3 3 6 4 5 4 4

6 5 2 6 5 5 5 6 6 6 4 3 5 4 6 6 3 5

6 6 1 2 4 6 2 4 5 6 5 2 6 6 2 2 1 4

4 5 5 3 4 6 6 1 5 6 4 3 4 6 4 5 6 4

4 6 4 4 5 6 6 2 5 6 6 4 4 6 6 3 5 4

6 5 2 2 3 4 6 2 3 6 6 6 1 6 2 6 4 2

6 4 6 3 6 4 6 2 6 6 4 1 5 6 6 3 4 6

6 6 6 6 6 6 6 1 5 6 3 6 6 4 6 2 2 6

5 6 5 6 4 5 6 1 6 6 5 5 4 4 6 6 6 5

4 6 3 6 6 5 5 3 4 6 3 4 6 6 5 5 5 4

6 6 4 6 6 6 6 1 6 6 6 6 4 6 5 6 6 6

4 4 3 1 6 4 6 1 4 6 6 4 4 4 5 6 3 4

4 2 4 6 4 5 5 4 5 6 4 5 4 5 4 5 6 4

6 6 6 4 6 6 6 4 6 6 3 6 6 6 6 6 6 6

5 5 3 4 2 5 5 3 3 6 5 1 6 6 3 4 1 4

2 5 3 3 3 5 6 2 5 6 2 2 4 6 3 3 3 1

5 4 4 6 6 6 5 6 5 6 3 5 2 6 6 5 5 6

3 6 2 4 4 5 5 4 5 5 4 4 4 6 5 4 5 5

4 4 3 4 5 5 5 3 3 5 4 4 4 5 4 4 4 4

3 5 2 3 5 3 6 4 1 5 6 3 2 6 6 5 4 3

5 5 3 4 5 5 6 1 6 6 6 5 5 5 6 5 6 1

4 5 5 6 4 5 6 4 3 6 3 4 6 6 6 4 5 2

5 3 2 2 4 6 6 1 3 6 4 4 4 6 6 5 5 5

6 5 4 1 6 6 5 1 6 5 6 5 6 5 3 6 5 1

5 5 5 6 5 5 4 6 6 6 2 5 6 1 6 1 5 6

4 6 5 6 5 6 4 4 6 6 5 6 6 5 5 3 6 6

5 4 4 6 6 5 5 3 6 5 4 5 5 6 4 5 4 4

5 6 2 6 4 4 6 5 4 6 4 4 5 5 4 5 2 3

5 5 2 3 4 5 6 4 5 6 4 4 3 6 4 4 5 3

2 6 6 2 5 3 5 2 3 6 4 2 5 6 4 5 5 6

5 6 5 4 5 5 6 3 4 5 4 4 5 6 5 6 5 4

5 6 4 5 5 5 6 3 2 6 4 3 3 6 5 6 4 5

3 5 5 5 6 5 6 4 5 6 3 5 6 5 5 6 5 6

4 6 5 6 6 5 6 5 4 6 5 6 6 4 6 5 5 3

4 6 1 4 5 5 6 3 3 6 2 4 4 6 5 5 6 4

5 6 1 6 6 5 5 3 5 6 6 6 6 6 3 6 6 5

4 6 4 6 5 6 5 4 5 5 4 5 6 6 5 5 6 3

6 6 3 5 6 5 6 3 4 6 3 6 6 5 4 6 2 5

5 5 3 5 5 5 4 4 5 6 4 5 6 4 4 4 5 4

4 6 3 6 3 5 6 3 5 6 4 4 4 6 5 6 5 5

5 5 2 2 4 5 5 2 3 5 3 2 2 5 3 5 4 5

5 5 5 6 5 5 5 3 6 6 3 5 6 4 5 4 3 4

5 5 1 5 6 6 6 6 6 6 6 6 2 6 4 5 6 2

6 6 1 6 6 6 6 5 5 6 5 6 6 6 6 6 6 5

6 5 3 4 3 4 6 3 5 5 5 2 4 5 2 4 3 3

4 5 1 4 4 5 5 2 5 6 5 5 4 5 5 6 4 2

5 5 3 4 4 5 6 3 5 6 6 5 3 5 6 5 5 6

4 4 4 6 4 4 5 4 6 6 6 6 4 6 4 6 6 6

5 6 3 4 5 5 6 2 6 6 6 2 4 6 6 6 3 6

6 4 4 5 4 5 4 3 3 5 5 5 4 3 3 5 4 6

6 6 3 4 5 5 5 5 5 6 6 5 4 6 5 5 3 5

5 6 3 3 4 5 6 3 5 6 5 6 4 6 3 6 3 2

5 6 2 4 5 5 5 5 5 5 3 4 4 4 5 3 4 5

4 6 5 6 6 5 4 4 5 6 6 5 5 6 5 6 6 6

3 6 5 5 4 5 5 4 4 6 3 3 1 2 1 5 3 6

5 6 5 5 5 5 6 5 6 6 6 6 5 6 5 5 4 5

6 6 3 5 5 5 6 1 4 6 4 3 4 6 5 4 3 5

5 6 4 5 5 5 4 3 3 6 5 4 3 5 6 5 5 5

5 6 5 5 3 3 5 3 6 6 5 6 6 5 5 5 5 6

4 5 4 5 5 4 4 5 4 5 5 4 5 4 5 5 6 6

6 6 2 5 6 4 6 3 4 6 5 4 5 6 4 6 3 6

6 4 4 5 6 5 6 2 4 5 5 4 4 4 4 5 6 5

6 6 4 6 6 5 4 4 5 6 5 5 5 5 4 6 6 4

4 5 5 5 5 5 6 5 5 6 4 4 5 6 5 4 3 4

6 6 2 5 6 5 6 4 5 6 5 6 3 6 5 6 5 6

6 6 5 4 5 5 6 2 6 6 6 2 5 6 6 6 5 5

5 5 3 4 5 6 5 3 3 6 5 4 4 5 4 5 5 4

2 5 3 2 5 5 6 3 3 6 5 3 5 5 4 4 5 5

4 5 3 5 5 5 6 6 4 5 4 4 6 6 4 6 4 6

6 5 4 6 6 5 6 3 4 6 4 4 3 4 5 5 6 6

5 5 3 5 4 5 4 3 3 5 5 4 3 4 5 5 5 4

6 5 3 4 6 5 5 5 5 6 4 5 4 4 5 4 4 6

6 6 6 6 6 6 6 5 6 6 5 3 3 6 6 6 2 1

2 6 5 5 6 6 6 4 5 6 4 5 4 5 4 5 5 5

4 5 4 5 6 6 6 6 5 6 5 5 6 4 6 5 5 6

4 6 5 6 6 6 6 3 3 6 6 3 4 6 6 6 4 3

4 5 3 6 3 5 5 4 6 6 6 5 6 5 6 5 4 4

6 6 2 5 5 5 6 2 3 6 5 5 3 6 5 5 6 1

6 5 4 5 2 5 6 2 5 5 5 5 5 6 6 5 6 5

5 6 4 6 6 5 6 4 4 6 3 5 6 6 5 6 4 6

4 6 2 3 6 4 5 1 6 6 4 2 2 5 3 6 6 3

3 5 2 4 3 5 5 1 3 3 3 3 3 5 3 4 4 3

5 6 4 6 3 5 6 6 5 6 5 6 5 6 5 6 4 5

2 5 2 2 3 5 3 3 4 4 4 2 4 5 5 4 5 1

6 6 3 6 6 6 6 1 6 6 3 6 5 5 5 6 6 6

6 6 1 2 3 5 5 3 2 6 6 2 2 6 2 6 5 2

3 5 3 3 5 5 6 2 5 5 4 3 3 6 5 4 3 3

6 5 3 1 5 5 6 3 5 6 6 5 4 6 4 6 6 4

4 6 2 3 6 4 5 4 5 6 6 2 4 6 4 6 4 5

5 5 3 4 2 4 4 1 4 4 3 3 3 5 4 4 4 3

6 6 1 6 6 5 6 6 4 6 2 3 6 6 6 6 6 6

6 6 1 6 4 4 6 4 5 6 6 3 3 6 5 5 4 4

6 5 1 3 5 5 6 5 5 6 6 5 2 6 6 4 5 4

4 5 3 3 4 5 5 2 4 6 3 3 3 6 4 5 3 4

5 6 4 6 5 6 6 2 6 6 5 6 2 5 6 6 6 2

4 4 4 3 4 5 5 3 6 4 5 5 6 5 1 4 5 4

5 6 3 6 6 5 5 3 5 6 6 4 3 6 4 6 4 6

5 6 1 2 6 6 6 2 6 6 6 1 6 6 6 6 6 6

5 6 2 5 5 6 6 3 6 6 2 1 1 3 5 6 2 4

4 6 1 6 4 5 6 3 6 6 5 5 4 6 6 6 6 6

5 5 5 4 4 5 6 3 4 6 4 3 4 6 4 5 6 4

4 6 4 5 5 6 6 1 4 6 6 4 4 6 6 6 6 6

3 5 4 5 4 6 5 3 5 5 4 3 4 5 6 4 3 4

5 6 3 4 6 5 6 1 5 6 4 3 4 4 5 5 3 4

5 5 2 3 1 5 5 5 6 6 4 3 5 5 6 5 6 6

4 5 2 5 5 5 5 3 5 5 3 4 5 6 6 5 2 6

5 5 5 4 5 2 6 2 6 6 1 6 6 6 6 4 4 6

4 6 3 1 5 5 6 6 6 6 6 3 1 6 4 6 4 1

4 6 4 4 5 5 5 4 4 6 3 3 5 6 5 6 6 5

6 6 1 5 4 5 6 3 5 6 3 4 6 5 6 3 5 6

4 5 3 3 2 4 4 3 4 5 4 4 4 6 4 5 2 4

2 5 4 6 4 4 5 1 4 6 3 3 3 6 5 4 6 2

5 4 4 6 5 5 6 2 6 6 3 5 6 1 5 5 4 5

5 5 4 3 2 6 5 1 5 6 6 3 4 6 2 5 6 2

6 5 2 4 6 5 6 1 6 6 4 6 4 6 5 4 6 2

6 5 3 2 6 4 4 1 6 5 4 3 4 5 6 6 4 6

5 6 3 2 5 6 6 2 6 6 4 3 3 5 5 5 5 4

6 6 5 5 6 6 6 6 5 6 1 2 6 6 2 6 6 5

5 5 5 5 5 6 6 3 3 6 5 3 5 6 5 5 4 5

5 6 4 4 5 6 6 1 5 6 6 3 3 6 4 6 3 5

6 5 2 5 5 5 6 2 4 6 5 5 3 6 4 5 3 5

5 5 1 5 5 5 6 3 5 6 2 3 5 6 5 5 6 5

2 6 2 2 4 6 6 1 5 6 6 3 3 5 6 6 6 6

6 5 1 2 5 5 6 1 2 6 5 2 3 5 5 5 5 6

4 5 3 3 5 5 6 1 4 6 4 4 2 6 4 5 2 2

6 6 3 1 6 4 5 1 4 6 4 1 3 6 2 6 6 2

4 4 3 4 2 6 4 1 2 5 4 2 6 5 6 5 1 2

6 6 2 5 6 4 2 4 6 6 6 6 6 6 2 5 5 1

5 6 2 2 4 6 5 3 5 6 5 4 5 6 5 2 3 4

3 5 2 3 5 5 6 1 2 6 5 3 5 6 4 5 5 5

6 6 5 6 1 6 6 5 5 6 5 2 6 6 6 6 6 6

5 6 4 4 4 4 5 2 4 3 5 5 5 6 5 5 3 4

5 5 3 5 5 5 5 2 5 6 5 2 3 6 4 6 5 4

5 5 3 4 5 5 5 2 5 6 4 3 5 6 4 6 3 4

5 6 2 6 6 6 6 4 5 5 5 5 6 6 4 5 3 5

6 5 2 5 5 5 6 2 4 6 5 5 3 6 5 5 4 4

3 5 4 5 6 5 6 4 5 5 2 3 5 5 4 4 4 5

4 5 2 4 6 5 6 4 5 5 4 4 2 5 1 5 4 2

5 1 3 4 6 5 5 3 3 4 5 3 4 6 5 4 5 5

4 6 2 5 6 5 5 1 6 6 4 5 4 6 5 6 6 5

4 6 1 3 2 5 6 4 5 5 6 5 3 5 5 5 5 5

4 6 2 3 5 5 4 1 6 6 6 3 6 6 6 6 6 4

3 4 2 4 5 4 6 3 5 6 4 5 4 6 3 5 6 5

3 5 2 3 3 4 3 3 3 6 6 6 5 6 4 3 2 1

2 6 4 3 2 5 6 1 6 6 3 1 4 3 5 4 5 1

4 6 6 5 6 4 6 3 5 6 5 3 6 4 6 6 6 6

6 6 2 6 6 5 4 6 6 6 5 6 5 6 6 6 5 2

5 5 2 4 4 5 3 2 5 5 4 5 4 5 4 4 4 3

3 6 1 1 3 5 6 4 5 6 1 4 3 6 4 6 1 4

5 6 3 1 6 6 4 3 6 6 3 5 6 6 6 6 6 3

1 6 4 4 4 5 6 1 6 5 5 3 3 4 6 5 4 1

4 6 5 4 2 6 6 2 5 6 6 3 3 6 6 6 6 3

5 5 3 6 4 5 2 3 6 6 5 3 6 4 5 5 4 5

4 4 2 6 4 6 6 5 4 6 6 6 4 6 6 6 6 6

4 4 3 3 4 6 6 4 3 5 4 3 3 5 4 6 3 4

5 6 3 2 5 5 6 2 6 5 4 5 5 6 4 4 4 6

6 6 4 6 6 6 6 4 5 6 5 4 5 6 6 6 5 6

2 3 3 2 5 4 3 1 5 5 6 1 2 5 4 2 5 3

4 6 5 2 6 6 6 6 4 5 1 1 2 6 5 5 6 5

5 5 5 6 6 6 6 2 5 6 4 5 5 6 6 3 6 6

5 6 1 3 6 6 6 1 6 6 5 3 3 6 6 6 6 6

5 4 2 3 5 3 5 5 4 6 3 4 3 6 5 4 5 3

4 5 3 5 1 5 6 4 5 6 1 4 2 5 6 5 5 5

6 5 4 5 3 5 6 3 5 6 6 5 3 5 6 6 4 6

5 6 4 4 2 3 5 6 5 6 6 5 5 4 4 5 5 4

4 5 3 6 4 5 6 2 3 4 3 3 3 6 4 4 3 3

4 6 1 4 5 5 4 3 4 6 4 3 2 6 6 6 6 6

4 6 3 4 5 4 5 4 5 5 5 5 5 5 5 5 4 5

6 6 2 1 6 6 6 6 6 6 4 6 3 6 6 6 3 4

6 6 1 4 6 6 6 3 2 6 6 6 3 6 6 6 5 6

6 6 3 1 5 5 5 1 6 6 3 3 3 6 2 5 3 5

6 6 2 3 3 5 5 3 6 5 5 4 3 6 4 4 4 2

5 5 2 5 5 4 5 2 5 5 3 3 5 5 5 3 2 5

6 6 2 1 3 4 3 2 3 5 4 2 1 6 5 6 5 4

5 6 3 5 5 5 4 1 3 6 5 3 5 6 4 5 4 3

5 5 2 6 4 6 3 1 6 6 6 5 4 5 6 5 6 6

3 6 4 4 4 5 5 4 4 6 4 2 3 6 3 5 5 3

4 6 1 4 5 5 4 5 5 5 4 5 3 6 4 5 6 3

6 5 1 5 5 5 6 2 5 5 5 5 5 3 6 5 3 5

5 5 1 5 5 6 6 1 3 6 4 4 3 5 4 4 4 5

4 6 4 5 2 5 6 6 4 6 4 6 3 5 6 6 5 6

5 5 1 5 5 6 6 3 4 1 3 4 4 5 4 4 5 4

5 6 2 2 3 6 4 1 6 5 4 4 3 6 1 5 1 1

6 6 1 2 6 5 6 4 6 6 5 3 4 6 6 6 4 6

6 5 3 5 6 6 5 1 4 6 3 4 5 5 4 4 5 6

5 5 4 4 3 5 5 4 3 5 4 4 3 6 4 5 5 3

4 5 2 4 4 5 4 2 4 6 4 6 3 6 6 5 6 4

6 6 6 6 6 6 6 1 6 6 5 1 6 5 6 6 6 6

6 6 2 4 3 4 5 1 6 6 6 3 4 6 4 5 5 6

5 6 1 3 6 3 4 1 5 6 6 6 3 5 1 6 6 1

4 5 4 6 5 5 6 1 4 6 4 4 2 6 2 1 3 3

3 5 3 3 4 4 5 4 5 6 4 5 4 5 4 5 5 3

6 5 1 2 4 5 5 4 3 6 6 2 2 5 5 6 5 5

4 5 2 3 4 5 5 2 4 5 4 5 5 6 5 3 5 5

5 4 1 4 3 5 6 2 6 6 6 3 3 4 6 6 6 6

5 5 2 4 5 4 6 1 3 6 2 3 1 6 2 6 2 2

5 5 3 5 6 6 6 5 5 6 4 4 5 6 6 4 5 5

5 6 4 3 3 5 6 4 4 4 3 4 4 6 4 4 3 4

5 5 3 3 3 6 6 1 4 6 4 4 4 6 4 5 5 5

4 5 3 5 4 5 6 2 5 6 5 4 4 6 3 4 4 4

6 5 2 3 4 5 5 5 4 6 4 5 2 5 2 4 5 3

6 6 3 4 5 6 6 4 6 5 6 3 2 5 3 5 6 3

6 3 4 4 4 5 3 2 4 6 4 3 4 5 4 5 5 4

2 6 6 3 2 4 6 1 5 6 3 3 5 6 3 4 3 2

3 5 4 4 5 5 6 5 6 6 5 4 2 6 3 4 5 5

5 6 4 5 2 4 1 4 5 6 4 4 3 6 5 4 5 3

5 6 4 6 4 6 6 5 6 6 4 4 4 5 5 5 6 5

LOADING VALUES

Items: 18

Values:

0.2406

0.1623

0.3350

0.7964

0.4853

0.2483

0.1862

0.4868

0.3712

0.1487

0.0382

0.4475

0.6330

0.0036

0.6234

0.2521

0.2345

0.7304

ML TRAIT ESTIMATES

Participants: N=346

Values:

1.9829

-0.8278

0.1143

-0.6591

0.8865

0.7031

1.2757

1.0328

-0.0510

1.4330

-1.2369

1.1120

1.4656

-0.1346

1.0849

0.1800

-0.5347

1.5608

0.0836

0.5336

-0.6262

0.8990

-1.0633

-0.0952

0.1806

0.5158

0.5370

0.2532

-0.0305

-1.0514

1.5885

0.4787

0.3908

1.2482

0.6838

1.3556

-0.7979

-2.7906

1.1630

0.1765

-3.3275

0.0031

0.5126

1.8603

-1.2788

-0.6419

-0.4157

0.9350

1.1910

0.0619

-1.0226

1.7053

-0.8816

-1.6210

2.6080

0.2207

2.2855

-1.5914

-0.4118

-1.4984

-1.0622

-5.0145

-0.9080

-0.9257

-1.1570

-1.6719

0.2598

-0.5795

0.9417

-0.3207

-0.1057

1.0384

1.2460

-1.1989

0.3198

-0.0445

0.9405

0.6385

0.1645

-1.3152

0.9096

-0.6734

1.1361

0.5890

-0.8800

0.5914

-0.7457

0.2616

-0.2283

0.5782

-0.0795

-1.9109

-0.1619

-0.0542

-1.4430

0.4666

0.9173

0.0699

0.3122

-0.3536

0.6271

-0.3107

-1.0893

-0.0531

0.5223

-2.9165

0.2288

0.4545

1.3567

0.8234

1.1641

1.0435

-1.1760

1.7854

-1.4330

0.9365

-1.9423

0.1362

-0.8306

-0.8657

-0.5778

1.1341

-1.7327

1.0121

2.3962

0.8675

2.4699

0.8858

-1.2405

-1.0297

0.4625

1.0511

0.3333

-0.0010

-1.8621

0.2683

-0.4722

1.7814

0.4748

-1.1063

1.4695

-1.5538

-0.4572

0.3295

-2.5223

0.3853

1.9755

1.3782

1.0218

1.9138

-1.1287

0.1446

2.5161

-1.3195

-2.2536

1.4572

-0.2046

-0.9208

-1.8016

0.0870

0.5726

-0.6361

-0.8647

1.7910

1.8977

0.6751

0.0598

-0.9224

-0.8401

0.4238

0.1408

1.6496

1.6816

-0.1489

1.1771

1.1164

1.1585

0.5224

0.6764

-2.0834

1.1640

0.2172

2.3633

-1.6712

-0.6578

0.5132

0.9017

0.6513

-0.2952

0.6270

-0.7511

0.0609

1.7342

-1.0713

1.5318

0.1592

0.3648

1.1069

0.5870

0.7316

0.2388

1.2288

0.6652

1.2611

1.0177

-0.2496

-0.8801

0.9174

1.0857

-0.5601

0.8398

0.9497

0.9030

2.0996

0.8217

1.0631

-0.4493

0.6342

1.9105

-1.4023

-2.5369

1.4807

-2.6129

2.0647

-2.6465

-1.2426

-0.4598

-0.3702

-2.1541

2.0678

0.0922

-0.1556

-1.2565

0.8251

-1.1292

0.8102

0.7653

-0.2709

1.3599

-0.2191

1.0927

-0.0439

0.0014

0.2316

0.6249

0.9943

-1.6360

0.5938

1.1187

-1.6369

-0.9569

1.3180

-1.6795

0.0499

-0.1362

-0.3455

1.2566

0.6926

-0.0544

-0.0414

0.6157

-0.3106

-1.0152

-1.6604

-2.2170

-1.4543

-0.2559

-0.5971

-0.8443

1.6990

-0.6141

-0.3242

-0.1615

1.2670

0.0400

0.3137

-1.6581

-0.8328

0.8068

-0.6642

0.2621

-0.4209

-2.0677

-1.6613

1.7004

1.4891

-1.0601

-1.5157

0.4865

-1.1265

-0.1866

0.6960

1.4226

-1.1465

-0.0419

2.2223

-3.0485

-0.3177

1.8068

0.5456

-1.0864

-0.1259

0.8188

-0.1384

-1.2056

-0.0307

0.2916

0.4753

0.8249

-1.4096

-1.3769

-0.2605

-2.4337

-0.4926

1.0098

-1.1411

-0.5879

0.6597

-0.4142

0.9511

-0.9213

-2.7426

0.5994

0.6786

-1.0880

-0.2501

2.1489

-0.2866

-1.9703

-1.4185

-0.8523

-1.1485

-0.5720

0.0657

-2.3209

1.4212

-0.9993

-0.5570

-0.5160

-1.4865

-0.5526

-0.8445

-1.7146

-0.3619

-0.8446

1.4190
