# Supplementary material for: Practical Person-Fit Assessment with the Linear FA Model: New Developments and a Comparative Study
Source: Front Psychol. 2016 Dec 27;7:1973. doi: 10.3389/fpsyg.2016.01973 (PMC5186803; doi:10.3389/fpsyg.2016.01973)
Supplement: Supplementary file 2 [file DataSheet2.DOC]

function out = Lico(X, t, lam)

%%%%%%%%%%%%%%%%%%%%%%%%%%%%%%%%%%%%%%%%%%%%%%%%%%%%%%%%%%%%%%%%%%%%%%%%%%%%%%%%%%%%%%%%%%%%%%%%%%%%%%

% Help for Lico.m

% PERSON FIT INDEX: LICO

%

% This index is a weighted mean-squared statistic which has unit expectation under the null hypothesis

% of inconsistency as proposed by Ferrando, Vigil-Colet & Lorenzo-Seva (2017). Large values suggest

% inconsistency: values of about 1.5 are generally used for judging potential inconsistency.

%

% INPUT

%

% X Matrix with responses related n participants to m items (n x m)

% t Latent scores estimates for n individuals in a single factor (n x 1)

% lam Loading coeficients of m items related to a single factor (m x 1)

%

% OUTPUT

%

% out Vector with Lico index for each participant (n x 1)

%

% DATE WRITTEN: 01 june 2016. Lorenzo-Seva, U.

% LAST UPDATE : 24 november 2016.

%

%%%%%%%%%%%%%%%%%%%%%%%%%%%%%%%%%%%%%%%%%%%%%%%%%%%%%%%%%%%%%%%%%%%%%%%%%%%%%%%%%%%%%%%%%%%%%%%%%%%%%%

[n,m] = size(X);

mu = mean(X);

S = cov(X);

out = zeros(n,1);

vare = diag((S-lam*lam'));

deno=sum(vare);

for i=1:n,

Lin = 0;

for j=1:m,

p2 = (X(i,j)-mu(j)-(lam(j)*t(i)));

Lin = Lin + (p2*p2);

end;

in= Lin/deno;

in=(m/(m-1))*in;

out(i)=in;

end;

return;

function out = rpg(X)

%%%%%%%%%%%%%%%%%%%%%%%%%%%%%%%%%%%%%%%%%%%%%%%%%%%%%%%%%%%%%%%%%%%%%%%%%%%%%%%%%%%%%%%%%%%%%%%%%%%%%%

%

% Help for rpg.m

%

% PERSON FIT INDEX: PERSONAL CORRELATION

%

% This index is the personal correlation as proposed by Fowler (1954) and Donlon & Fisher (1968).

% It is computed as the correlation between the respondent’s response vector and the vector of

% item sample means.

%

% INPUT

%

% X Matrix with responses related to n participants to m items (n x m)

%

% OUTPUT

%

% out Vector with personal correlation for each participant (n x 1)

%

% DATE WRITTEN: 01 june 2016. Lorenzo-Seva, U.

% LAST UPDATE : 24 november 2016.

%

%%%%%%%%%%%%%%%%%%%%%%%%%%%%%%%%%%%%%%%%%%%%%%%%%%%%%%%%%%%%%%%%%%%%%%%%%%%%%%%%%%%%%%%%%%%%%%%%%%%%%%

[n,m] = size(X);

mu = mean(X);

out = zeros(n,1);

for i=1:n,

r = corrcoef(X(i,:),mu);

out(i)=r(1,2);

end;

return;
